# Evaluation of Immunofluorescence Antibody Test Used for the Diagnosis of Canine Leishmaniasis in the Mediterranean Basin: A Systematic Review and Meta-Analysis

**DOI:** 10.1371/journal.pone.0161051

**Published:** 2016-08-18

**Authors:** Amel Adel, Dirk Berkvens, Emmanuel Abatih, Abdelkrim Soukehal, Juana Bianchini, Claude Saegerman

**Affiliations:** 1 Institute of Veterinary Sciences, University Saad Dahlab, Blida, Algeria; 2 Institute of Tropical Medicine, Department of Biomedical Sciences, Antwerpen, Belgium; 3 Research Unit of Epidemiology and Risk Analysis applied to veterinary science (UREAR-ULg), Fundamental and Applied Research for Animals & Health (FARAH) Center, Faculty of Veterinary Medicine, University of Liege, Liege, Belgium; 4 University Hospital of Beni Messous, Algiers, Algeria; Universita degli Studi di Parma, ITALY

## Abstract

With an expected sensitivity (Se) of 96% and specificity (Sp) of 98%, the immunofluorescence antibody test (IFAT) is frequently used as a reference test to validate new diagnostic methods and estimate the canine leihmaniasis (CanL) true prevalence in the Mediterranean basin. To review the diagnostic accuracy of IFAT to diagnose CanL in this area with reference to its Se and Sp and elucidate the potential causes of their variations, a systematic review was conducted (31 studies for the 26-year period). Three IFAT validation methods stood out: the classical contingency table method, methods based on statistical models and those based on experimental studies. A variation in the IFAT Se and Sp values and cut-off values was observed. For the classical validation method based on a meta-analysis, the Se of IFAT was estimated in this area as 89.86% and 31.25% in symptomatic and asymptomatic dogs, respectively. The Sp of IFAT was estimated in non-endemic and endemic areas as 98.12% and 96.57%, respectively. IFAT can be considered as a good standard test in non-endemic areas for CanL, but its accuracy declines in endemic areas due to the complexity of the disease. Indeed, the accuracy of IFAT is due to the negative results obtained in non-infected dogs from non-endemic areas and to the positive results obtained in sera of symptomatic dogs living in endemic areas. But IFAT results are not unequivocal when it comes to determining CanL infection on asymptomatic dogs living in endemic areas. Statistical methods might be a solution to overcome the lack of gold standard, to better categorize groups of animals investigated, to assess optimal cut-off values and to allow a better estimate of the true prevalence aiming information on preventive/control measures for CanL.

## Introduction

Leishmaniases are parasitic diseases ranked second in mortality and fourth in morbidity among tropical diseases, with about 2 million disability-adjusted life years [1]. It was also considered of high importance in a recent prioritization process based on a multi-criteria decision making which involved 100 food producing animal diseases and zoonoses [2]. In humans, the disease occurs in four main forms: cutaneous, diffuse cutaneous, mucocutaneous and visceral [3]. The latter is caused by *Leishmania infantum* and is characterized by irregular bouts of fever, substantial weight loss, swelling of the spleen and liver, and anemia. If left untreated, the fatality rate within two years can be as high as 100% in developing countries [4–5].

In the Middle East, Mediterranean countries, Iran, Pakistan, Afghanistan, Brazil, and China, visceral leishmaniasis (VL) is a zoonotic disease transmitted by female sand flies belonging to the genus *Phlebotomus* [6]. In the Mediterranean basin, the incidence risk of VL is relatively low, ranging from 0.07 to 1.6 cases per 100,000 inhabitants [7–9]. Despite this relatively low incidence risk, the health impact of VL is severe. Moreover, the disease is spreading to regions previously considered as non-endemic [10]; probably because of climate change, human made changes and population movements [7].

Dogs are considered the major host for this parasite and the main reservoir for human infection [11]. The clinical symptoms and time of appearance of canine leishmaniasis (CanL) in dogs vary widely from apparently healthy to critically diseased [12–14]. This is dependent on the balance between cellular and humoral immune responses [15–16].

Since both symptomatic and asymptomatic dogs are infectious to sand fly vectors, this allows the transmission of the parasite to other dogs and humans [17–19]. Therefore early detection and treatment of infected animals is the best way to reduce the risk of infection and is an essential part of the prevention and control of the disease in humans [20–21] and, as such, a prime example of the “One Health” concept.

Epidemiological studies on CanL are regularly conducted with the aim to estimate the true prevalence of the disease. It is in this regard important to note that the characteristics of the diagnostic technique(s) used may have a considerable influence on the estimate obtained for the true prevalence [22–23]. Sensitivity (the conditional probability that the test yields a positive result given the individual is truly a case) and specificity (the conditional probability that the test yields a negative result given the individual is truly a non-case) are indicators of the diagnostic test validity [24–25]. An effective diagnosis test should have high analytic and diagnostic sensitivity (Se), analytic and diagnostic specificity (Sp), repeatability and reproducibility, a defined threshold (cut-off) and a good fitness for the intended purpose(s); it must be simple, easy to perform, non-expensive, feasible in regional laboratories or adaptable for field conditions [20, 26].

According to the World Organisation for Animal Health [23], serology is the preferred diagnostic method for CanL and VL, even during the early stages of the disease. So, with a 96% Se and a 98% Sp, the indirect fluorescent antibody test (IFAT) is one of the most suitable diagnostic tests. Despite the fact that IFAT is not a perfect test, it is frequently used as a reference test for the relative validation of new diagnostic methods [27–32] and to estimate the true CanL prevalence [33–35]. To assess the reliability of a new test in comparison with IFAT, the kappa coefficient is often utilized (e.g. [30, 32, 36–37]). However, a limitation of the kappa test is that it is affected by the prevalence of the condition under observation [38] and, thus, it is possible that despite a high concordance between two tests, the kappa coefficient may paradoxically be low. Expressing concordance between test results in terms of indices of positive and negative agreement is the preferred alternative to the kappa coefficient [39].

It is important here to be precise about the so-called gold standard (perfect test): it is a test or procedure that is absolutely accurate, *i*.*e*. it diagnoses all of the specific diseased individuals that exist and misdiagnoses none [25]. However, a gold standard test is quasi non-existent in veterinary medicine and it must be understood that a reference test (high Se and Sp not necessary equal to 100%) is not a gold standard (Se and Sp = 100%). Accuracy assessment of diagnostic tests may be seriously biased if an imperfect reference test is used [40]. Sensitivity and specificity are population parameters that describe the test performance for a given reference population. However, it is a common observation that Se and Sp estimates vary among published validation studies [41–42].

The aim of the present study was to conduct a systematic literature review on the accuracy of IFAT for the estimation of CanL prevalence in the countries of the Mediterranean basin. In addition, we sought potential causes of variation in the Se and the Sp of IFAT among different epidemiological surveys with the aim to better inform preventive and control measures for CanL. Finally, to estimate the Se and Sp of IFAT in different contexts, a meta-analysis was conducted based on selected available classical studies.

## Materials and Methods

### Systematic review

**Fig 1:**
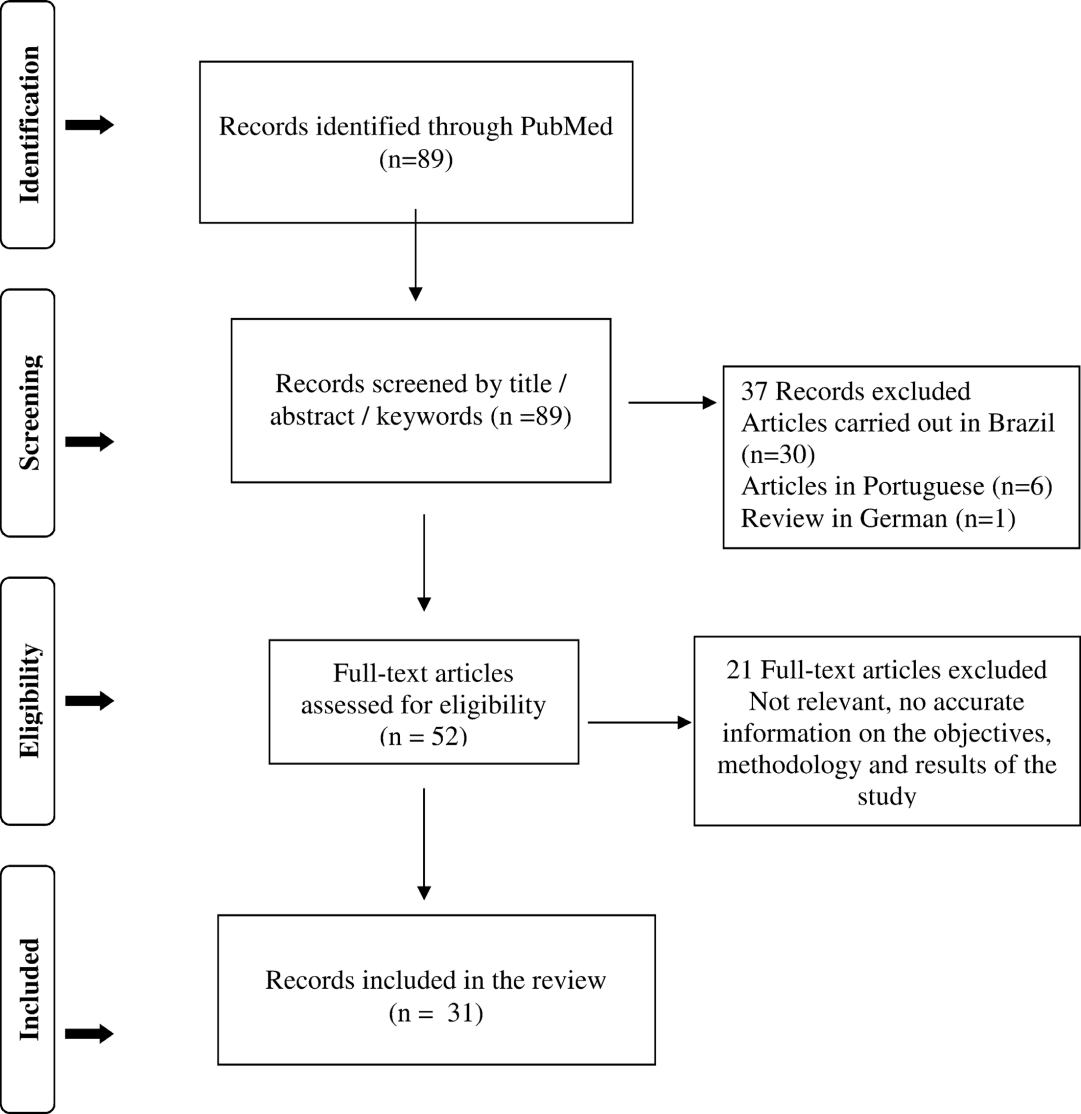
The Preferred Reporting Items for Systematic Reviews and Meta-Analyses (PRISMA) Flow Diagram. To the left are different steps of the PRISMA flow diagram; to the right are the results obtained after each of these steps.

This systematic review was conducted in the PubMed database on all papers published before July 31, 2014 (period of 26 years, 1988–2014). With the aim to ensure rigorous and transparent reporting, the Preferred Reporting of Systematic Reviews and Meta-Analysis (PRISMA) guidelines [43–44] were applied (**Fig 1**). Studies were identified using a combination of keywords. The terms searched for were (insensitive): (canine leishmaniasis) AND (immunofluorescence antibody test OR ifat OR ifa OR ifi) AND (diagnostic accuracy OR validation OR sensitivity OR specificity).

First, we checked the lists of titles and abstracts identified by this search to determine the relevance of the articles. Literature review studies were left out. We also excluded studies conducted in countries other than the Mediterranean basin and studies using IFAT as a gold standard to assess other diagnostic tests.

The selected articles were read in full to confirm eligibility. Thereafter, the following data were extracted and gathered into a structured table: reference, objectives of the study, type of study, study period, area of origin of the dogs under study, sample size, inclusion criteria for dogs, type of IFAT used (in-house or commercial), test threshold, type of validation and, lastly, the main findings related to IFAT.

### Meta-analysis

Meta-analysis used to determine diagnostic test accuracy is a statistical technique, which combines findings from independent studies [45]. A meta-analysis was performed based on available studies generated with classical validation methods (i.e. using a reference test) and aiming the estimation of the Se of IFAT in symptomatic and asymptomatic dogs originating from endemic Mediterranean basin. In the same manner the Sp of IFAT in non-endemic and endemic area was estimated. The Se or Sp was calculated in different contexts from studies reporting a single proportion (results of systematic review) using meta, an R package for meta-analysis ([46]; R-3.0.1, R Foundation for Statistical Computing, http://www.r-project.org/). A random effects meta-analysis model was used in order to better account for heterogeneities (within and between study variability) among the different studies considered [47].

## Results

### Systematic review

Of the 89 articles flagged by the search related to the validation of IFAT, 21 did not meet the aim of this review, 30 were carried out in Brazil, 6 were in Portuguese and one literature review study was in German. Thus, a total of 31 articles met the inclusion criteria (see validation of the indirect fluorescent antibody test (IFAT) for canine leishmaniasis in the Mediterranean basin) for the 26-year period available.

Validation of IFAT in the 31 selected articles was conducted both north and south of the Mediterranean basin (**S1 Table**). Dogs sampled in the studies were from both endemic and non-endemic regions and sample sizes ranged from 22 to 1,035 dogs. Furthermore, the IFAT accuracy was explored in three groups: non-infected dogs, subclinically infected dogs and diseased dogs. The IFAT threshold varied from 1/20 to 1/200 with a mode of 1/80. In one study, the IFAT cut-off was not mentioned [48].

All the included studies were based on an *in-house* IFAT, with the exception of one study that used a commercial kit [49].

Three methods of validation were used: classical contingency table analysis (2x2: IFAT *versus* parasitological examination and/or culture and/or immunoblotting and/or polymerase chain reaction test, PCR) (26 studies), statistical validation (2 studies) and experimental validation (3 studies).

#### Classical contingency table analysis

The so-called classical validation was the most frequently used (26 articles) including 22 studies that used parasitological examinations and/or culture as reference test and 4 other studies that used as reference test respectively an immunoblotting and PCR [50], a western blot technique [51], a standard blood PCR [52] and a PCR-Enzyme-Linked Immunosorbent Assay (ELISA) [22].

Among these 22 studies, four studies established IFAT as 100% sensitive and 100% specific irrespective of the dog’s clinical status [53–56].

Although Otranto et al. [57], Mancianti et al. [58–59] and Mettler et al. [60] reported 100% specificity; they found also lower sensitivity values, respectively 99%, 98.4%, 98.7% and 90%.

In addition, IFAT Se decreased down to 29.4% in asymptomatic dogs from 90% in symptomatic diseased dogs [61]. Moreover, only 2 dogs out of 22 asymptomatic dogs were IFAT positive whereas 12 out of 13 affected dogs had an IFAT titre above the cut-off value (>1:100) [51].

Some studies did not estimate Se and/or Sp but showed a discrepancy between the results of IFAT (positive or negative) and the reference test used [28, 37, 50, 61–63].

Furthermore, a study carried out to evaluate the serological cross-reactivity between *Leishmania* and other canine pathogens, showed that out of 57 dog samples tested, 11 tested falsely positive for IFAT [64]. However, in Tunisia, and among 250 asymptomatic dogs tested for leishmaniasis, 9 dogs were positive to IFAT and were confirmed by at least one other method in one or more tissues (direct examination, culture, PCR) [65].

#### Statistical validation

In the statistical validation, two types of techniques were used: the latent class analysis (LCA) and the Bayesian approach [40, 66].

Validity analysis for three CanL serological tests including IFAT with respect to parasitology and disease, were compared with latent class analysis [40]. This survey was carried out on 151 stray dogs of Tunisia. The analysis was based on the method proposed by Qu et al. [67] and compared three constructed latent class analysis models. IFAT was found to be 100% sensitive and 100% specific in a two latent classes (infected, non-infected) model including a conditional dependence [68] between clinical definition and parasitology in the group of infected dogs. On the other hand, IFAT was 100% sensitive and 93.6% specific in the classical validation against the parasitological examination.

A Bayesian approach was used to evaluate three serological tests including IFAT for CanL in three groups of dogs according to their functional type (stray dogs, farm dogs and national guard dogs) in Algeria [66]. The analysis showed that IFAT was definitely not a gold standard: the sensitivity was respectively 94.7%, 94.9% and 89.7% in the three groups and the specificity varied from 65.2% in the farm dog group to 94.5% for stray dogs.

#### Experimental validation

Experimental validation of IFAT was assessed using naturally infected dogs, i.e. with positive culture of lymph nodes or bone marrow aspiration or chancre biopsies [69] or using healthy dogs that were subsequently experimentally infected by *Leishmania* [70–71]. In the first study, Se and Sp with respect to infection can be simultaneously high, but maximum sensitivity is probably <80% and it lasts for a relatively short period of 2–3 months after a lengthy incubation period [69]. For the two other studies [70–71], respectively 63% and 65% Se and 82% and 94% Sp were obtained. However, the small number of papers does not allow for having conclusive results. Moreover, two studies were done on only 6 dogs [70, 71], whereas the third study [69] had a bigger number of dogs (N = 50).

### Meta-analysis

**Fig 2:**
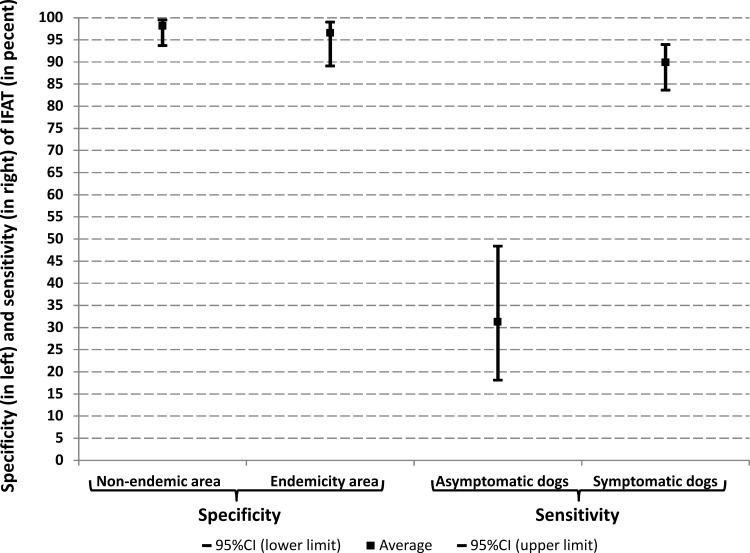
Estimated sensitivity and specificity of IFAT based on the systematic review and meta-analysis (based on available studies from classical validation method). The specificity was estimated in non-endemic and endemic area. The sensitivity was estimated in asymptomatic and symptomatic dogs in endemic Mediterranean basin. The sensitivity of the IFAT in endemic areas was estimated using a sum of 103 asymptomatic dogs (32 of them were positive in IFAT) and using a sum of 173 symptomatic dogs (161 of them were positive in IFAT). The corresponding numbers of Se studies for asymptomatic and symptomatic dogs were [22, 50, 53, 60, 81] and [22, 28, 50, 52, 53, 54, 60, 81] respectively. The corresponding numbers of Sp studies for non-endemic and endemic area were [53, 54, 60, 76] and [48, 54, 55, 57, 58, 59, 76, 80] respectively (see **S1 Table**). Heterogeneity tests were significant (p = 0.01 and p<0.001, for the Se, and the Sp respectively).

For classical validation method only and based on the selected available studies in the Mediterranean basin after systematic review, the sensitivity of IFAT was estimated using a random effects meta-analysis in symptomatic and asymptomatic dogs (**Fig 2**). The Se of IFAT was estimated to be 89.86 (95% CI: 83.63–93.89) and 31.25% (95% CI: 18.09–48.35) for symptomatic and asymptomatic dogs respectively. In addition, the Sp of IFAT was estimated as 98.12 (95% CI: 93.69–99.46) and 96.57 (89.06–98.98) in non-endemic and endemic areas respectively.

## Discussion

### Systematic review

The ideal way to evaluate a diagnostic test is to compare its results with those of a definitive error-free reference test or gold-standard [72]. In the present study, the focus was on three alternative ways of diagnostic test validation: the classical, the statistical and the experimental validation methods.

*Classical validation method*. IFAT assessment was often based on a comparison to a reference test, mostly to a parasitological examination. Unfortunately, direct microscopic observation of stained smears or indirect culturing of tissue fragments or aspirates are highly specific but not very sensitive [52, 73]. Indeed, these comparisons showed a variation in the effectiveness of IFAT from high to very low values of its Se and Sp. Several factors come to mind to explain this variation (see below). According to Greiner and Gardner [41] and Saegerman et al. [42], the reference populations, sampling strategies, stage duration of infection, threshold and the area under investigation are possible factors responsible for Se and Sp variation.

*Sample size*. IFAT owes its status of gold standard to Mancianti and Meciani [53] who found it to be 100% sensitive and 100% specific in detecting *L*. *infantum* antibodies in dogs with severe clinical signs, mild signs and without signs of disease, against parasitological culture of lymph node biopsies as reference test. Nevertheless, they stated that IFAT could fail in the early stages of the disease when IgG are not yet detectable. As a consequence, the reduction of its sensitivity to 98.4% and 98.7% was reported in later studies by the same authors [58–59]. Moreover, this study was carried out on small sample sizes with 52 infected dogs and 36 control dogs. Consequently, these two parameters had wide 95% confidence intervals. Detecting Immunoglobulin M (IgM) antibodies that appear early in the course of an infection, could be an alternative. However, the evaluation of an ELISA based on anti-dog IgM showed a low accuracy (Se = 23% with a 95% CI between 11% and 46%; Sp = 99% with a 95% CI between 88% and 100%) [70]. Moreover, when assessing IFAT anti-*Leishmania* IgM in cats, this test was found to be 100% specific but not suitable for epidemiological surveys because of its low Se [74].

*Cross-reactions*. Sp is decreased by cross-reactions [41–42]. For this reason, the abovementioned validation studies used groups of dogs with other diseases but negative for *L*. *infantum*, but the group sizes were small, respectively 21, 47 and 11. A number of different parasitological diseases (dirofilariosis, borreliosis, cryptococcosis, babesiosis and ehrlichiosis), as well as chronic pyoderma, chronic hepatitis and renal disorders were considered and no cross-reactions were observed. The absence of cross-reactions between *L*. *infantum* and *Ehrlichia canis* was later also found by Liéra et al. [75].

In contrast, Otranto et al. [76] reported one false positive result in a dog from Apulia, Southern Italy, an endemic area where canine monocyclic ehrlichiosis was diagnosed. False positive reactions using IFAT were observed with *Trypanosoma cruzi* [77], *Ehrlichia canis* [78], *Demodex canis* [79] and *Toxoplasma gondii* [64] in Brazil. Of these, *T*. *cruzi* is the only parasite that is not an issue in the Mediterranean basin, being exotic to this area.

*Reference populations*. With respect to reference populations, IFAT was shown to be 100% sensitive and 100% specific when used on clinically suspected expatriate dogs [55]. The characteristics of IFAT were calculated with reference to the combined serological positivity of the micro-immuno-diffusion and immune-electrophoresis. These two tests are not known to be gold standard tests for CanL. Moreover, these results cannot be extrapolated to the target population of CanL. Indeed, IFAT’s Se changes from 100% to 80.5%, when it is assessed in dogs from non-endemic areas, that occasionally visit endemic areas, and in dogs residing in endemic areas [54].

Also, in a longitudinal survey carried out in Southern France, an endemic area for CanL, IFAT Se and Sp were estimated at 84.6% and 76.5% respectively [80].

*Clinical status*. CanL has a disease spectrum in which clinical disease represents one pole of the infection and asymptomatic subclinical infection the other [81].

Thus, IFAT is a good test when used in a group of symptomatic dogs (Se = 90 to 100%, Sp = 100%), but its efficiency is markedly lower when the dogs are asymptomatic, having therea Se of 29.4% [60]. Also, when compared to PCR and immunobloting, only one out of seventeen asymptomatic PCR positive dogs was IFAT positive [50]. Similarly, a study carried out in an endemic area (Athens, Greece) on dogs suspected of leishmaniasis, which compared IFAT to the standard blood PCR yielded 82 PCR negatives against 86 IFAT negatives. The four IFAT negative results were attributed to an immunodeficiency and the resulting inability to produce sufficient amounts of antibodies. On the other hand, the observed discrepancy between PCR and IFAT positive cases (65 positives by PCR against 74 IFAT positives) was explained by antibodies persisting even after the elimination of PCR detectable *Leishmania* DNA [52]. Iniesta et al. [82] also reported a lack of specificity in IFAT, noting a very poor performance when having to discriminate between uninfected and infected asymptomatic dogs. A study in Alto Douro (Portugal), where IFAT had 97% sensitivity irrespective of clinical signs [83], suffered from small sample size (33 symptomatic dogs and only one asymptomatic dog).

In addition, the detection of the T-cell mediated immunity against leishmaniasis in asymptomatic dogs showed an increase of the prevalence of infection, compared with those obtained by IFAT. Hence, when combining IFAT with a leishmanin skin test (LST) in parallel, 27 dogs were considered positive out of 58 asymptomatic dogs (using IFAT alone yielded 15 positive dogs [81]). According to Martin-Sanchez et al. (2001), a PCR-ELISA combination also had a higher sensitivity than IFAT [84].

When compared to positive results obtained by PCR in blood, nested-PCR (nPCR) in bone marrow or conjunctival swab (CS), the number of positive dogs detected by IFAT ranged from none to 50% [28, 37, 63]. A possible explanation for these discrepancies could be a difference in the duration of infection. Antibody development in infected animals can take from months to years [85], whereas CS PCR shows positive results within 6 weeks after infection [86]. Regarding discordance with IFAT, the number of seronegative dogs detected as positive by CS n-PCR (n = 16) which is similar to the number of seropositive dogs detected as negative by CS n-PCR (n = 15) probably reflects the inherent limits of both tests in detecting different stages of infection [37].

*Life cycle stage*. Variation in Se and Sp was shown to be dependent on the life-cycle stage (amastigotes versus promastigotes) [51]. Indeed, all animals with clinical manifestations had titres above the cut-off value (1:100) in the IFAT using amastigotes as antigen whereas only one affected dog had a titer of 1:50 in the IFAT when using promastigotes as antigen. However, only one study cannot allow to draw potent conclusions.

*Cut-off value*. Test-results are dependent on the diagnostic cut-off value [41]. The cut-off value for a serologic reaction is the result of a compromise between Se and Sp desired for the test. Lowering the cut-off value increases test Se and correspondingly decreases Sp [87–88]. Furthermore, a valid threshold for a sample of the population is not necessarily valid at individual level. A lower cut-off titre may reveal early or subclinical disease [80].

By comparing antibody titres against *L*. *infantum* in a group of dogs sampled *at random* (DSR) and in a group of dogs with symptomatology compatible with CanL (DSCCanL), both the DSR and the DSCCanL groups produced high percentages of animals with doubtful antibody titres (61.2% and 22.5% respectively, when the threshold was 1:160) [22].

Therefore, the dependence of the diagnostic Se and Sp on the selected cut-off value must be considered for a full test evaluation and for test comparisons; these problems might be addressed by the receiver-operating characteristic (ROC) analysis [89]. The threshold could also be determined using the Bayesian Markov chain Monte Carlo mixed-model [90]. In this case, the observed data are separated in two distributions assumed to represent negative and positive individuals. A new test threshold is selected for the target group of animals based on the fitted distributions.

#### Statistical validation method

A full explanation of the statistical methods in use is found in [64, 91]. The studyby Boelaert et al. [40] used the latent class analysis and Adel et al. (63) a Bayesian approach. With the former analysis, IFAT was found to be a gold standard (Se and Sp were estimated as 100%). It should be noted that in this case the number of dogs investigated was limited and the animals were all stray dogs. These dogs are known to be an easier target for infection and sand fly biting due to the outdoor living habits and precarious physical conditions [33, 92].

Adel et al. [64] found IFAT to be highly sensitive and highly specific in stray dogs, but not of gold standard quality. It was also found considerably less specific in farm dogs (65.2%). A similar dependence on type of dog was also found by Morales-Yuste et al. [22], who showed that a positive result in two serological techniques (including IFAT) for the same animal is 4.8 times more likely in guard dogs than in dogs kept as pets. This shows a variation in IFAT Se and Sp related to dog’s function and therefore its lifestyle habits.

#### Experimental validation method

The “gold standard” status of IFAT was challenged by experimental studies [69–70]. Their results clearly highlight the need to revise the status of IFAT as a gold standard for the diagnosis of CanL. In these two studies respectively, IFAT was 63% and 65% sensitive, and 82% and 94% specific.

Experimental conditions are of course not comparable to natural field infections since the outcome of *Leishmania* infection in animal models will depend not only on host immunity but also on a combination of factors, such as inoculated species, virulence of the strain, nature of the inoculum, number of parasites and route of inoculation [93].

Another finding related to another carefully observed cohort of naturally infected dogs is a seasonal variation in IFAT Se and Sp. For example, monthly changes in Se and Sp were shown by Dye et al. [68]. This study revealed that while Sp was always high, Se rose slowly at the end of the first year of the follow-up transmission season, taking 8 to 9 months to reach a peak. This occurred in March (Se = 86%) and April (Se = 83%) of the second year when using a threshold of ≥1:40. This implies that in an environment where transmission is highly seasonal, sero-epidemiological studies attempting to estimate the true prevalence of infection would have to be very carefully timed. The decline of sensitivity was explained by the group of positive animals that sero-reverted. This is in agreement with the 35% of hunting dogs with a positive titre (>1:80), which decreases to 0 at the end of the monthly follow-up study of the antibody titres in southern Spain, indicating a remission of the infection and explains the presence of dogs with a cellular immune response [15]. As already explained a humoral reaction is not protective and development of cellular immune protection is accompanied by a declined serotitres.

### Meta-analysis

For classical validation method only and even though the difference estimated Sp in non-endemic and endemic areas was not statistically significant, this study revealed that the estimation of Se in endemic Mediterranean basin was quite different in function of the symptomatic status of the dogs. Only 2 studies estimated IFAT Se in non-endemic areas and found it 100% [54, 55].

A discrepancy in estimating sensitivity and specificity of IFAT was also demonstrated in another meta-analysis study carried out in Brazil [94]. In fact, the combined results of 11 studies on IFAT provided an estimate of 88% for the sensitivity (95% CI: 85–91) and 63% for the specificity (95% CI: 61–65). Furthermore, a subgroup analysis of the influence of the pre-screening selection strategy showed an overestimation of the specificity in healthy dogs from non-endemic areas. The moderate Se (72–100%) and Sp (52–100%) of IFAT used in the Brazilian ministry of Health in its dog screening-culling campaigns was invoked as one of the reasons of low effectiveness of the campaigns [95].

## Conclusion

We have outlined several issues involved in IFAT validation. In the classical validation, we observed that IFAT was validated against parasitological exams as reference tests, known not to be very sensitive, hence hampering the validity of the analysis. Throughout the selected studies a great variation in the IFAT sensitivity and specificity values was observed in function of many parameters (see **Table 1**). IFAT can be considered as a good standard test in non-endemic areas for CanL, but its accuracy declines in endemic areas due to the complexity of the disease. In other words, the accuracy of IFAT is due to the negative results obtained in non-infected dogs from non-endemic areas and to the positive results obtained in sera of symptomatic dogs living in endemic areas. But IFAT results are not unequivocal when it comes to determining CanL infection on asymptomatic dogs living in endemic areas. Indeed, both symptomatic and asymptomatic dogs represent a risk of infection to other dogs and humans and IFAT shows considerable difficulties detecting asymptomatic dogs (see **Fig 2**). Additionally, these studies do not always describe the target population and do not follow proper sampling methods sometimes employing very small sample sizes.

**Table 1:** Parameters that might influence IFAT sensitivity and specificity.

Parameters	Epidemiologic methods recommended
Sample size	Random sampling and estimation of correct sample size
Cross-reactions	ROC curve / Latent class analysis
Reference populations	Multi-testing / Bayesian approach
Clinical status	Accurate case definition
Season effect	Repeat testing
Life cycle stage	Good fitness according to the purpose
Cut-off value	ROC curve / Latent class analysis

ROC: Receiver Operating Characteristic.

A better definition of the target population is crucial for a better estimation of the prevalence of CanL. In the absence of a single accepted reference standard for a specific target condition, it may be possible to construct one, based on two or more tests. A pre-specified rule to determine the individual status must be established based on specific combinations of results [96].

More widespread use of statistical methods and more specifically latent class methods offers a possible way to deal with the lack of a gold standard, to both better categorize groups of animals under investigation and to obtain optimal cut-off values allowing a better estimate of the true prevalence. Indeed, the World Assembly of delegates of the OIE recently added the Bayesian approach to the OIE terrestrial manual [26]. However, and because these models are complex, statistical assistance will be required to describe the sampling of the target population, the characteristics of the tests in use, and the appropriate model [26, 91].

## Supporting Information

S1 Table Validation of the indirect fluorescent antibody test (IFAT) for canine leishmaniasis in the Mediterranean basin. Table with all studies retained in the systematic review. CanL: Canine leishmaniasis; IFAT: Immunofluorescence antibody test; Se: Sensitivity; Sp: Specificity; Inf: Infected; NI: Not infected;E: Endemic; NE: Non endemic; ELISA: Enzyme linked immunosorbent assay; IHAT: Indirect hemmaglutination; CIEP: Counterimmunoelectrophoresis; DAT: Direct agglutination test; PCR: Polymerase chain reaction; LST: Leishmanin skin test; LAMP: Loop mediated isothermal amplification of DNA, CS: Conjunctival swab.
(DOCX)
