# Supplementary material for: Evaluation of Immunofluorescence Antibody Test Used for the Diagnosis of Canine Leishmaniasis in the Mediterranean Basin: A Systematic Review and Meta-Analysis
Source: PLoS One. 2016 Aug 18;11(8):e0161051. doi: 10.1371/journal.pone.0161051 (PMC4990183; doi:10.1371/journal.pone.0161051)
Supplement: S1 Table — Table with all studies retained in the systematic review. CanL: Canine leishmaniasis; IFAT: Immunofluorescence antibody test; Se: Sensitivity; Sp: Specificity; Inf: Infected; NI: Not infected;E: Endemic; NE: Non endemic; ELISA: Enzyme linked immunosorbent assay; IHAT: Indirect hemmaglutination; CIEP: Counterimmunoelectrophoresis; DAT: Direct agglutination test; PCR: Polymerase chain reaction; LST: Leishmanin skin test; LAMP: Loop mediated isothermal amplification of DNA, CS: Conjunctival swab. (DOCX) [file pone.0161051.s001.docx]

**S1 Table.** Validation of the indirect fluorescent antibody test (IFAT) for canine leishmaniasis in the Mediterranean basin

| Reference | Objectives | Study period | Type of study | Area of origin | Sample size | Inclusion criteria | IFAT  test | Threshold | Mode of validation | Main findings related to IFAT |
| --- | --- | --- | --- | --- | --- | --- | --- | --- | --- | --- |
| 1. **Classical contingency validation** | | | | | | | | | | |
| [53] | To evaluate three serotests (IFAT, IHAT, CIEP) | Not listed | Not listed | Endemic (Italy) | 52 infected (Inf) dogs  36 control dogs(NI) | Inf: 26 with severe signs, 15 with mild signs and 11 asymptomatic  NI: 15 healthy and 21 with leptospirosis, toxoplasmosis, nephropathy, dermatitis | In house | 1:40 | *Versus* popliteal lymph node biopsy + culture in modified Toby medium | IFAT:  Se=100%, Sp=100% |
| [61] | To compare DAT with IFAT, ELISA, cross-over electrophoresis and the latex agglutination test | Not listed | Cross-sectional | Endemic (France) | 152 dogs | 34 dogs with positive lymph node aspirates | In house | 1:160 | *Versus* parasitology exam of lymph node aspirates | DAT more sensitive than IFAT (Se=82.35%)  24 positive to DAT of which 16 positive to IFAT had not been parasitologically confirmed |
| [58] | To compare *L. infantum* promastigotes antigen ELISA with IFAT | Not listed | Not listed | Endemic (Tuscany, Italy) | 290 dogs | 186 *Leishmania* infected dogs  104 control dogs | In house | 1:40 | *Versus* parasite cultivation and by isolation inoculating hamsters | IFAT:  Se=98.4%, Sp=100% |
| [50] | To investigate performance of PCR and immunoblotting for detection of asymptomatic dogs | Not listed | Cohort | Endemic (Marseille, France) | 58 dogs | *At-random* selected dogs: 18 with acute leishmaniasis  10 treated dogs with previous disease  30 asymptomatic dogs (aged 3to 1é months) | In house | 1:50 | *Versus* immunoblotting and PCR | Symptomatic dogs: Se= 100%  Asymptomatic dogs: 1 IFAT positive out of 17 immunobloting and PCR + |
| [59] | To compare Dot-ELISA and IFAT | Not listed | Not listed | Endemic (Isle of Elba, Italy) | 149 infected dogs  75 non-infected  11 NI with other diseases (cryptococcosis, ehrlichiosis, chronic pyoderma, chronic hepatitis) | Inf: symptomatic, oligosymptomatic and asymptomatic dogs | In house | 1:40 | *Versus* Culture of lymph node aspirates | IFAT:  Se= 98.7%  Sp= 100% |
| [54] | To compare IDA to IFAT and ELISA | Not listed | Cohort | Endemic (Naples, Italy)  And non-endemic (Holland) | 52 infected dogs  75 negative controls | Inf: 44 endemic and 8 non endemic  NI: 40 from Naples, 35 from Holland (20 diseased and 15 healthy) | In house | 1:80 | *Verus* clinical signs, direct observation of the parasite or positive to IFAT | Endemic symptomatic and parasite +: Se=93%, Sp=100%  Symptomatic and parasite -: Se=88%,  Sp= 100%  Symptomatic expatriate: Se=100%  Sp=100% |
| [55] | To develop a slide ELISA and compare it to 4 tests | Not listed | Not listed | Non endemic | 32 dogs suspected of leishmaniasis  8 dogs with heterologous diseases (Babesiosis, Ehrlichiosis, Dirofilariosis, Borreliosis and Trypanosomosis) | Clinically suspected Belgian dogs known to have travelled to France, Spain or Portugal | In house | 1:32 |  | IFAT Se=100%, Sp=100% |
| [81] | To apply the leishmanin skin test (LST) to the detection of *Leishmania* infection in dog | Not listed | cross-sectional | Endemic  (Alto Douro, Portugal) | 58 asymptomatic dogs  4 symptomatic dogs | Asymptomatic with no history of leishmaniasis | In house | 1:20 | *Versus* NNN culture of bone marrow | IFAT + LST: 27 positive dogs out of 58  IFAT alone: 15 positive dogs out of 58 and 4 positive out of 4 dogs  Culture: 1 symptomatic positive, 3 symptomatic positives |
| [80] | To assess the Latex particle agglutination test | Not listed | Longitudinal  (second control of positives after one month) | Endemic (Pyrénées Orientales, Southern France) | 1035 dogs | Hunting, guard, farm and pet dogs | In house | 1:160 | *Versus* NNN culture of lymph nodes aspirates | Se= 84.6%  SP= 76.5% |
| [51] | To compare IFAT using promastigotes or amastigotes in the diagnosis of CanL | Not listed | Cross-sectional | Madrid, Spain | 35 dogs | 22 asymptomatic dogs  13 naturally infected dogs | In house | 1:100 | *Versus* Western Blot technique | IFATa is equally specific but more sensitive than IFATp |
| [84] | To describe PCR-ELISA technique and to correctly meaning titres | Not listed | Not listed | Endemic (Granada, Spain) | 31 dogs | Destined for euthanasia | In house | 1:80 or 1:160 |  | PCR-ELISA has a higher sensitivity than IFAT  17 IFAT positive against 26 positive PCR-ELISA on peripheral blood |
| [75] | To develop a mixed IFAT for canine ehrlichiosis and leishmaniaisis | Not listed | Cross-sectional | Madrid, Spain | 160 dogs | From veterinary clinics | In house | 1:160 |  | Absence of cross-reactions between *E. canis* and *L. infantum* |
| [56] | To report on the development and standardization of rk39 ELISA | Not listed | Not listed | Endemic (Italy) | 380 infected dogs |  | In house | 1:80 | *Versus* microscopy of bone marrow and lymph node aspirates | IFAT:  Se=100%  Sp=100% |
| [52] | To identify a reliable molecular tool for leishmaniasis diagnosis | Not listed | Cross-sectional | Endemic (Athens, Greece) | 160 dogs | Suspected of leishmaniasis or positive to IFAT | In house | 1:200 | *Versus* standard PCR assay on blood samples | 65 positives by both methods against 74 IFAT positives  82 negatives by both methods against 86 IFAT negatives |
| [57] | To evaluate the performance of rk39 rapid immunochromatographic test and IFAT | Not listed | Not listed | Endemic (Apulia, Southern Italy)  Non-endemic (Sweden) | 108 dogs | 68 infected endemic dogs  40 non endemic dogs (22 healthy and 18 with Sarcoptic mange, ehrlichiosis, borreliosis) | In house | 1:80 | *Versus* microscopy of lymph node aspirates smears | IFAT:  Se= 99%  Sp=100% |
| [62] | To extend the application of rk39 –ELISA in dipstick format in Turkey | 1996-2000 | Cross-sectional | Endemic (low endemicity) (Turkey) | 22 dogs | Clinically suspected dogs from veterinarian clinics or from field studies | In house | 1:128 | *Versus* microscopy of bone marrow and lymph node aspirates | One IFAT positive but negative to microscopy |
| [60] | Evaluation of ELISAs compared with IFAT and two rapid tests in symptomatic and asymptomatic dogs | Not listed | Cross-sectional | Non-endemic  (Southern-Zurich, Switzerland)  Endemic (Mediterranean areas) | 171 dogs | 48 stray dogs (non-endemic)  47 infected dogs (endemic)  50 healthy dogs (clinic of vet faculty of Zurich)  26 dogs with other parasite infections (*Babesia canis*, *Toxoplasma gondii, Neospora caninum*, *Hepatozoon canis*) | In house | 1:40 | *Versus* PCR of lymph node aspirates and/ or *in-vitro* cultivation | In Asymptomatic dogs Se= 29.4%  In symptomatic Se= 90%  Sp=100%  No cross-reactions |
| [76] | To validate the rk39 dipstick test for CanL | Not listed | Not listed | Endemic (E) (Apulia, Southern Italy)  Non-endemic (NE)(Sweden) | 165 dogs | 33 E negative (asymptomatic, no history of CanL and parasitology negative)  22 healthy NE  68 parasite positive symptomatic  24 E with other diseases  18 NE with other diseases | In house | 1:80 | *Versus* lymph node microscopic examination | IFAT :  Se=98.53%  Sp=98.97% (canine monocyclic ehrlichiosis) |
| [48] | To analyse the humoral immune response against *L. infantum* | Not listed | Not listed | Endemic (Morocco) | 81 dogs | 31 infected dogs  50 healthy negative controls | Not listed | Not listed | *Versus* parasite observation and/or after NNN culture | IFAT:  Sp= 94% |
| [65] | To assess the CanL prevalence | April to June 2003 | Longitudinal  (second control after 8 months) | Low-endemicity area (Sfax, Tunisia) | 9 IFAT positives out of 250 dogs investigated | Dogs from rural districts | In house | 1:40 | *Versus* Smear, culture, PCR | All IFAT positive cases were confirmed by at least one other diagnostic method |
| [83] | To evaluate the exo-antigen based ELISA | Not listed | Cross- sectional | Endemic (Alto Douro, Portugal) | 34 dogs | 33 symptomatic and one asymptomatic | In house | 1:80 | *Versus*  parasitological exam of bone marrow biopsies | IFAT:  Se= 97% irrespective of clinical signs |
| [63] | To assess the use of parasitological, seroogical and molecular methods for detection of *L. infantum* in blood | June and July 2005 | Random cross-sectional | Low-endemicity area (Kairaouen, Tunisia) | 67 dogs (24 symptomatic and 43 asymptomatic) | Outdoors dogs in rural distric | In house | 1:80 | *Versus* total number of samples positive with at least one method (PCR, IFAT, *in vitro* culture) | 21% of dogs were PCR positive  12% were IFAT positive with 4% of them positive to parasitology  IFAT:  Se = 50% |
| [37] | To evaluate the diagnostic performance of conjunctival swab (CS) nested-PCR in comparison to IFAT, lymph node microscopy and buffy coat (BC)n-PCR | November 2008- September 2009 | Cross-sectional | Endemic  (Umbria and Marche-Central Italy) | 253 dogs |  | In house | 1:160 |  | 18 positive dogs to n-PCR (16 CS and 2BC) were negative to IFAT  15 seropositive dogs were CS n-PCR negative |
| [22] | To establish the influence on the prevalence of CanL of: the use of different tests, different thresholds, selection of animals, function of dogs and *scenarii* with differing epidemiological characteristics | 2006-2007 | Cross-sectional | Endemic (Southeastern Spain, Granada, Spain)  Non-endemic (Starsbourg, France) | 502 dogs | 416 sampled at random in endemic area  71 symptomatic dogs  15 dogs via vet. Clinics in Strasbourg | In house | 1:80 or 1:160 | *Versus* PCR-ELISA | General data:  Se(1/80)= 38.5%, Sp=75.1% ;  Se(1/160)= 23.7%, Sp= 87.6%  Differences in Se and Sp according to presence of clinical signs, area under study and function of dogs |
| [49] | To develop a LAMP assay and compare it to IFAT, PCR and microscopic diagnostic | End 2008 -Beginning 2012 | Cross-sectional | Endemic  (Grand Tunis, Tunisia) | 75 symptomatic dogs (40 microscopy negatives and 35 microscopy positives) | Domestic dogs with visible clinical signs of leishmaniasis recruited at the national school of veterinary medicine clinics | commercial | 1:80 | *Versus* microscopy of smears of lymph nodes aspirates | IFAT sensitivity (Se) = 88.5% [74-95.4]  IFAT specificity (Sp) = 45% [34-51] |
| [28] | To evaluate a conjunctival swab PCR for the detection of *Leishmania* *infantum* in dogs in a non-endemic area | 2007-2011 | Cross-sectional | Munich, Germany | 74 dogs | 43 dogs defined as infected  37 with clinical signs  37 asymptomatic dogs | In house | 1:100 | *Versus* bone marrow PCR or lymph node aspirates PCR or blood PCR | Of 17 negative or doubtful by antibody testing (13 IFAT and 4 ELISA), 4 had a positive conjunctival swab PCR |
| 1. **Statistical validation** | | | | | | | | | | |
| [40] | To compare latent class analysis to classical validation of serological tests | Not listed | Cross-sectional | Endemic (Tunisia) | 151 dogs | Street dogs at risk of *L. infantum* | In house | 1:100 | *Versus* Latent class analysis  *Versus* classical validation (direct smear examination + culture) | Se= Sp= 100%  Se= 100%  Sp= 94% |
| [66] | To compare three serological tests (IFAT,DAT, PaGIA).  To assess the prevalence. | Oct 2004 -June 2005 | Cross-sectional | Endemic (Algiers, Algeria) | 462 dogs | Asymptomatic dogs  Stray dogs (S): 218  Guard dogs (G): 92  Farm dogs (F): 87 | In house | 1:128 | *Versus* Bayesian framework | (S) Se = 94.7%  Sp = 94.5%  (G) Se = 89.7%  Sp = 88.8%  (F) Se = 94.9%  Sp = 65.2% |
| 1. **Experimental validation** | | | | | | | | | | |
| [69] | To investigate performance of IFAT in detecting *L. infantum* infection | May 1989- May 1991 | Cohort (2 years) | Endemic (Cevennes, France) | 50 dogs | Dogs aged of 6 months naturally infected in Kennels with VL focus | In house | 1:40 or 1/80 | *Versus* infected population (positives to culture of lymph nodes or bone marrow aspirates or chancres biopsies ) | Se and Sp with respect to infection can be simultaneously high (two months) with max Se < 80% |
| [70] | To assess the operative validity of tests used in detection of *Leishmania* infection | Not listed | Longitudinal (one year) | Experimental infection | 6 dogs and 60 samples | Healthy, nine months old and kept indoor | In house | 1:80 | *Versus* experimental infection | IFAT:  Se=63% [43-79], Sp=82% [56-94]  Accuracy=68%[56-80] |
| [71] | To compare several commercially available tests for CanL diagnosis | Not listed | Longitudinal | Experimental infection | 6 dogs and 60 samples (19 from pre-infection phase and 41 from post-infection phase) | Healthy, nine months old and kept indoor | In house | 1:80 | *Versus* experimental infection | IFAT:  Se=65% [46-80], Sp=94% [72-99]  Accuracy=74%[62-85] |

Legend: CanL: Canine leishmaniasis; IFAT: Immunofluorescence antibody test; Se: Sensitivity; Sp: Specificity; Inf: Infected; NI: Not infected;E: Endemic; NE: Non endemic; ELISA: Enzyme linked immunosorbent assay; IHAT: Indirect hemmaglutination; CIEP: Counterimmunoelectrophoresis; DAT: Direct agglutination test; PCR: Polymerase chain reaction; LST: Leishmanin skin test; LAMP: Loop mediated isothermal amplification of DNA, CS: Conjunctival swab.
